# Pediatrics

**Published:** 1898-01

**Authors:** Maud Josephine Frye

**Affiliations:** Physician to the babies’ ward, Erie County Hospital


### Progress in Medical Science.

### PEDIATRICS.

Conducted by MAUD JOSEPHINE FRYE, M. D.,
Physician to the babies' ward, Erie County Hospital.

### PRE-NATAL INFECTION IN DISEASES OF INFANCY.

DAVIS (*Archives of Pediatrics*, September, 1897,) reports the result of investigations carried on for the past few years at the maternity department of the Jefferson College Hospital for the purpose of determining the cause of death in Winckel's disease. He says: With those who observe considerable numbers of infants, it is not uncommon to find cases in which a child, apparently healthy at birth, develops during the first few weeks of life a condition of apparent infection ending fatally. The predominant feature in these cases is the disorganised state of the infant's blood, manifested by hemorrhages from the bowels, sometimes from the vagina, urethra, nose, mouth and, in extreme cases, from the skin. Under the name of Winckel's disease, hemorrhagic diseases of the newborn, hemoglobinemia and other titles these cases have been variously described. In private practice, cases are not infrequently observed which do not go to the point of actual hemorrhage, but in which the newborn child gradually develops green stools, prostration, convulsions, coma and death. From analogy and from the successful treatment of some of these cases, we assign to them an infectious origin and separate them from cases of hemorrhage only by degree and not by kind.

The investigations warrant the following conclusions: No relation exists between the number of cells or percentage of hemoglobin in a given mother and the same in her infant. It cannot be said that the infant becomes hemorrhagic or develops toxemia because the mother is deficient in blood. Except in the acute infections, as typhoid and sepsis, the placenta is sterile. The feces of the infant before it has nursed may contain micrococci. In one case, terminating fatally, pyogenes albus, was obtained pure culture from the feces before nursing; a possible pre-natal infection. The mother's milk before the child has nursed may contain micrococci, affording in these cases a ready explanation of the infection. In conditions of maternal toxemia the same poison which affects the mother is transmitted to the child in some cases by substances other than bacteria.

Bacteriological investigation of three cases of hemoglobinemia discovered a germ, resembling that associated with yellow fever, present in the blood in severe cases which is capable of transmission through pregnant animals to their young.

The treatment most successful in these cases and that which the bacteriological examinations of the milk and feces show to be rational, is copious intestinal irrigation with normal salt solution. Calomel was of little value ; the ordinary laxatives, as castor oil, without result. Partially digested Pasteurised milk must in some cases be substituted for breast-feeding.

---

### ANEMIA FOLLOWING DIARRHEA.

JOHNSTON (*Archives of Pediatrics*, August, 1897,) calls attention to the obstinate anemia sometimes following diarrheal diseases. The digestion is weak, the appetite capricious, assimilation is imperfect. Such children fail to gain on cod liver oil and iron, as ordinarily given. A successful plan of treatment may be carried out by observing two points : to aid digestion and to give an easily assimilated form of iron. The writer has found rhubarb and soda for the digestive trouble, combined with pepto-mangan for the anemia, to act admirably in these cases, attention, of course, being given the diet.

---

### OPERATION FOR INTESTINAL OBSTRUCTION IN AN INFANT.

ROGERS (*Medical News*, October 2, 1897,) reports a successful operation for intestinal obstruction in an infant 64 hours old. Repeated doses of castor oil and intestinal irrigation had failed to move the bowels. During the thirteen hours preceding the operation fecal vomiting occurred three times. Just prior to the operation there was some distension of the abdomen, the pulse was 145, the child was sleeping quietly and the symptoms did not seem urgent. The abdomen was carefully washed with soap and water and sponged with a 1–60 carbolic acid solution, no scrubbing or hard rubbing being done. The cord was trimmed down as closely as possible and the stump carefully touched with a 1–20 solution of the same acid. An incision was then made in the median line, circling the umbilicus and extending as far as possible in both directions. At about its middle the small intestine was found closely bound down to the posterior portion of the cavity by a transverse band, which passed directly across, but was not adherent to the gut. This band appeared like the omental bands commonly seen leading into hernial sacs. It was ligated and the central portion cut away. The intestine above the obstruction was much distended; below, it was contracted to the size of a little finger. Considerable oozing took place, the blood being taken up on sponges. No flushing or irrigating was done. The incision was closed with silkworm gut sutures and a sterile dressing applied. The child was under the anesthetic thirty-five minutes. Seven hours after the operation the bowels moved. The child was stimulated by spartein, hypodermically, in doses ranging from 1-16 to 1-24 grains. The stitches were removed in a week, the wound being perfectly healed. The infant subsequently gained rapidly.

